# Supplementary material for: Effects of Dietary Alfalfa Meal Supplementation on the Growth Performance, Nutrient Apparent Digestibility, Serum Parameters, and Intestinal Microbiota of Raccoon Dogs (Nyctereutes procyonoides)
Source: Animals (Basel). 2024 Feb 15;14(4):623. doi: 10.3390/ani14040623 (PMC10886288; doi:10.3390/ani14040623)
Supplement: Supplementary file 1 [file animals-14-00623-s001.zip › animals-2842678-supplementary.pdf]

1 Supplementary Tables

Table S1. Effects of dietary alfalfa meal levels on serum immune indices of raccoon dogs (*n* = 7)

Item	Level of alfalfa meal, %				Mean	SEM	*P*-value
	0	5	10	15			
IgA, μg/mL	225.24	216.03	213.97	216.98	218.06	6.16	0.931
IgG, g/L	2.63	2.69	2.73	2.82	2.72	0.13	0.970
IgM, μg/mL	194.41	181.99	192.56	180.27	187.31	7.15	0.873

IgA = Immunoglobulin A, IgG = Immunoglobulin G, IgM = Immunoglobulin M.

Table S2. Difference in alpha diversity of intestinal flora of among four groups (*n* = 7)

Item	Level of alfalfa meal, %				Mean	SEM	*P*-value
	0	5	10	15			
Cecum							
Observed_otus	474.71	498.86	479.43	495.14	487.04	12.10	0.884
Chao1	476.48	503.73	481.27	501.64	490.78	12.21	0.826
Shannon	6.76	6.84	6.79	6.78	6.79	0.05	0.951
Simpson	0.977	0.978	0.979	0.979	0.979	0.001	0.814
Coverage	1.0000	0.9996	0.9999	0.9996	0.9998	0.0001	0.174
Colon							
Observed_otus	542.00^{b}	622.86^{ab}	464.14^{b}	736.14^{a}	591.29	33.32	0.018
Chao1	543.55^{b}	629.77^{ab}	466.17^{b}	742.66^{a}	595.54	33.47	0.015
Shannon	6.91^{b}	7.03ab	6..84^{b}	7.55^{a}	7.08	0.11	0.082
Simpson	0.977	0.976	0.980	0.984	0.979	0.002	0.454
Coverage	1.0000^{ab}	0.9996^{b}	1.0000^{a}	0.9992^{c}	0.9997	0.0001	<0.001

Ce = cecum, Co = colon, AM = alfalfa meal.

$^{a, b, c}$ With a row, values with different superscripts are significantly different ($P < 0.05$).

Table S3. The intestinal flora in phylum level (%)

Phyla	Ce.AM 0	Ce.AM 5	Ce.AM 10	Ce.AM 15	Co.AM 0	Co.AM 5	Co.AM 10	Co.AM 15
Bacteroidetes	76.82	69.67	74.55	73.99	79.37	56.26	67.47	57.30
Firmicutes	10.25	12.72	13.25	7.97	11.23	13.58	17.16	26.63
Fusobacteria	3.87	9.56	4.28	4.48	0.95	14.88	3.97	6.05
Proteobacteria	6.31	4.74	4.57	3.87	4.43	8.75	6.24	2.74
Spirochaetes	1.36	1.88	1.97	7.72	1.45	1.64	4.12	5.59
Actinobacteria	0.28	0.40	0.30	0.19	1.00	1.62	0.09	0.14

Ce = cecum, Co = colon, AM = alfalfa meal.

Table S4. The intestinal flora in genus level (%)

Genus	Ce.AM 0	Ce.AM 5	Ce.AM 10	Ce.AM 15	Co.AM 0	Co.AM 5	Co.AM 10	Co.AM 15
Prevotella	54.51	46.14	56.62	54.55	51.78	34.49	50.68	34.43
Alloprevotella	12.43	8.96	8.27	8.37	12.68	7.01	6.57	6.14
Fusobacterium	2.36	7.00	2.99	3.56	0.53	11.00	2.62	5.07
Bacteroides	2.08	4.60	1.86	1.66	2.79	4.09	1.64	1.86
Treponema	0.97	1.47	1.87	6.39	1.13	1.31	3.84	5.38
Muribaculaceae	0.66	2.29	0.66	0.82	3.21	3.25	0.86	3.29
Streptococcus	1.57	2.46	2.66	0.18	0.90	3.90	2.68	1.24
Sutterella	2.93	1.64	1.59	1.61	1.45	1.71	2.13	0.85
Sphaerochaeta	0.38	0.41	0.11	1.29	0.33	0.33	0.27	0.20
Anaerobiospirillum	1.58	1.50	0.85	0.54	0.95	2.24	2.77	0.41
Rikenellaceae_RC9_gut_group	0.81	0.94	0.74	1.25	0.81	0.76	0.65	2.07
Prevotellaceae_UCG-003	1.04	1.03	0.81	1.26	1.22	0.64	1.45	2.50
Succinivibrio	0.35	0.52	0.35	0.33	0.40	1.04	0.23	0.33
Clostridia_UCG-014	0.33	0.32	0.27	0.48	0.46	0.52	0.75	1.97
Phascolarctobacterium	0.20	0.47	0.32	0.03	0.22	0.76	1.14	0.94
UCG-005	0.15	0.18	0.17	0.24	0.94	0.11	0.42	1.20
Ruminococcus	0.17	0.13	0.12	0.32	0.17	0.12	0.20	1.18

Ce = cecum, Co = colon, AM = alfalfa meal.

2 Supplementary Figures

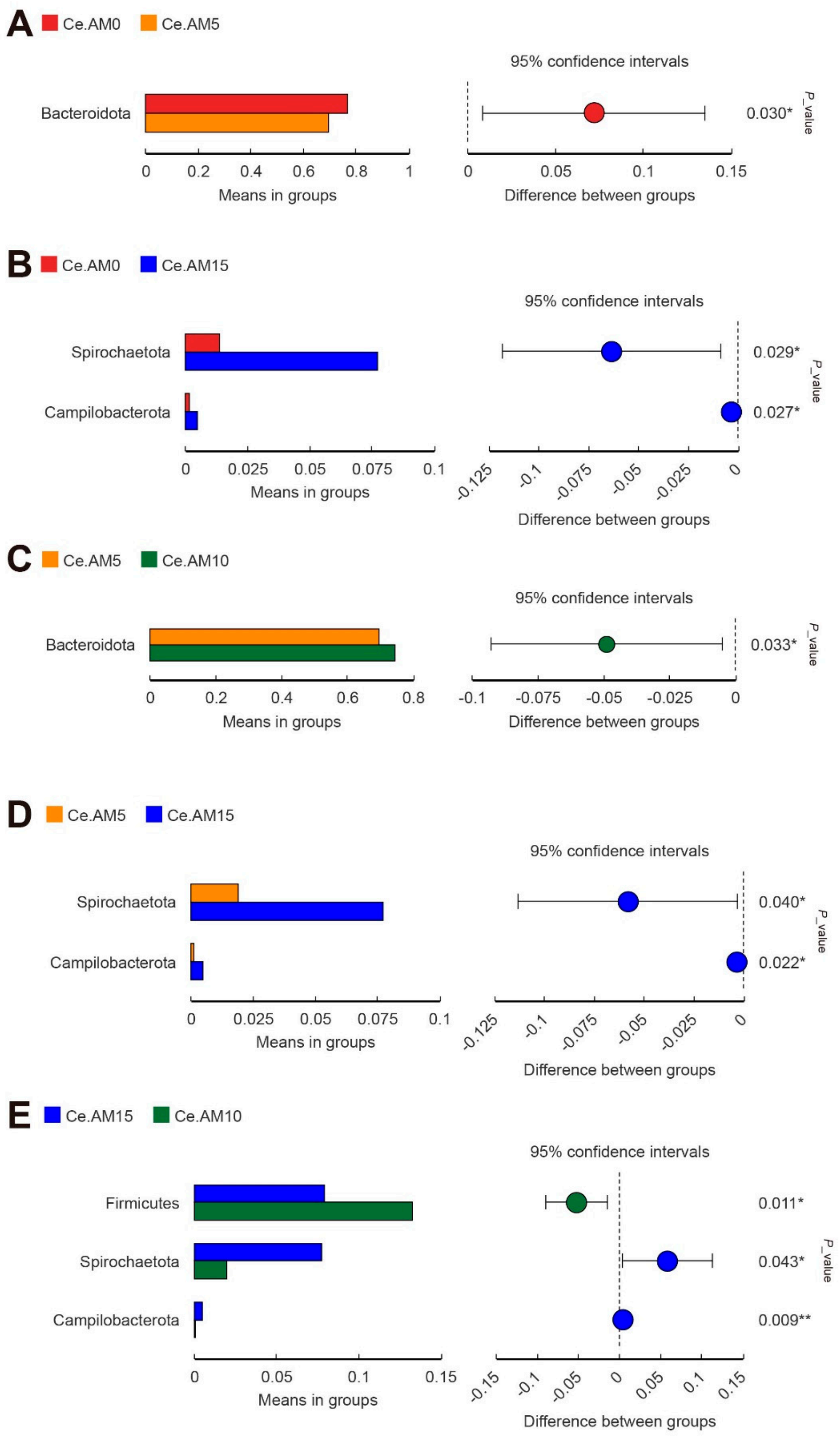

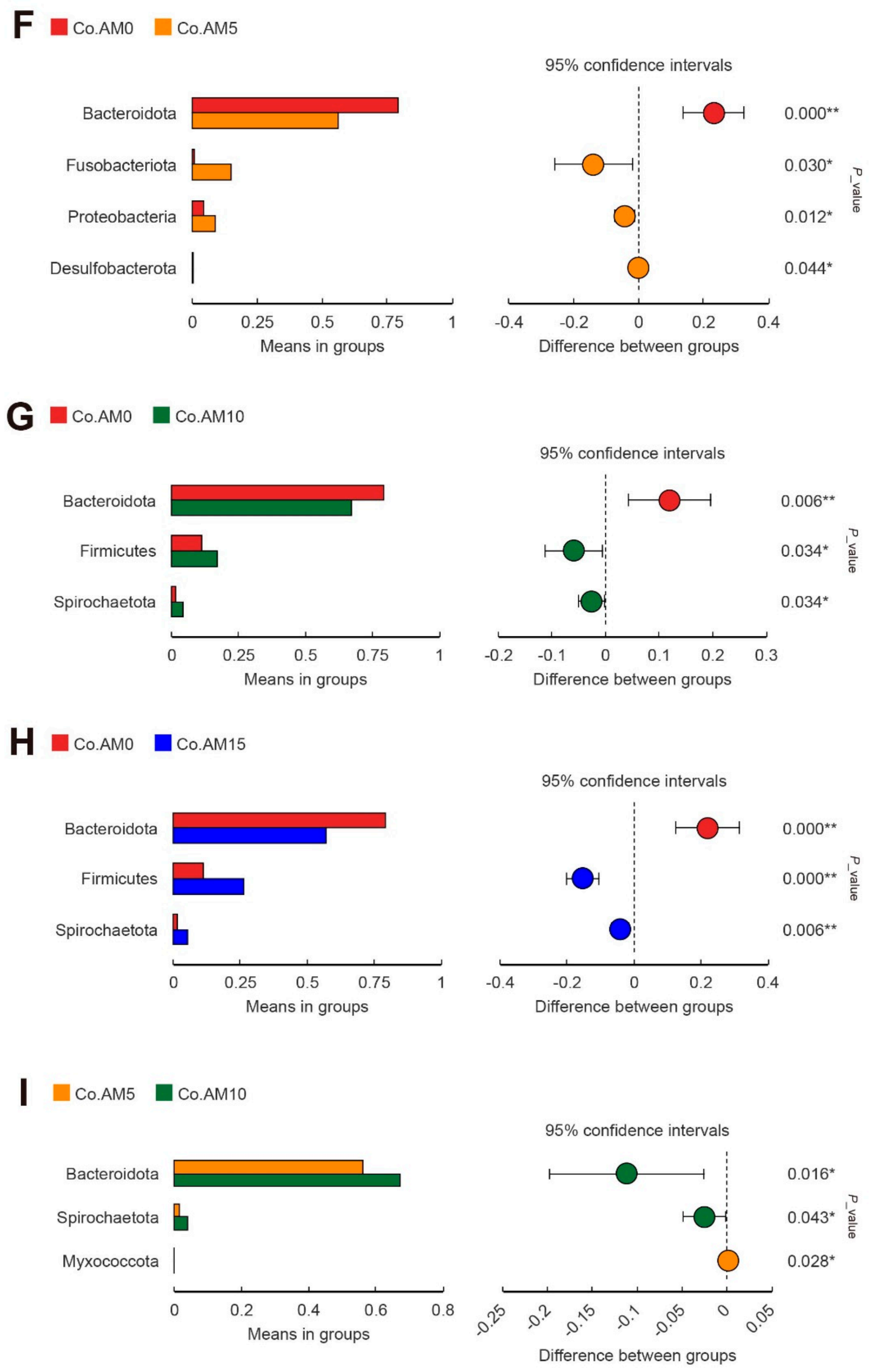
F
Co.AM0
Co.AM5
95% confidence intervals
Bacteroidota
Fusobacteriota
Proteobacteria
Desulfobacterota
0.000**
0.030*
0.012*
0.044*
P_value
0 0.25 0.5 0.75 1
Means in groups
-0.4 -0.2 0 0.2 0.4
Difference between groups
G
Co.AM0
Co.AM10
95% confidence intervals
Bacteroidota
Firmicutes
Spirochaetota
0.006**
0.034*
0.034*
P_value
0 0.25 0.5 0.75 1
Means in groups
-0.2 -0.1 0 0.1 0.2 0.3
Difference between groups
H
Co.AM0
Co.AM15
95% confidence intervals
Bacteroidota
Firmicutes
Spirochaetota
0.000**
0.000**
0.006**
P_value
0 0.25 0.5 0.75 1
Means in groups
-0.4 -0.2 0 0.2 0.4
Difference between groups
I
Co.AM5
Co.AM10
95% confidence intervals
Bacteroidota
Spirochaetota
Myxococcota
0.016*
0.043*
0.028*
P_value
0 0.2 0.4 0.6 0.8
Means in groups
-0.25 -0.2 -0.15 -0.1 -0.05 0 0.05
Difference between groups

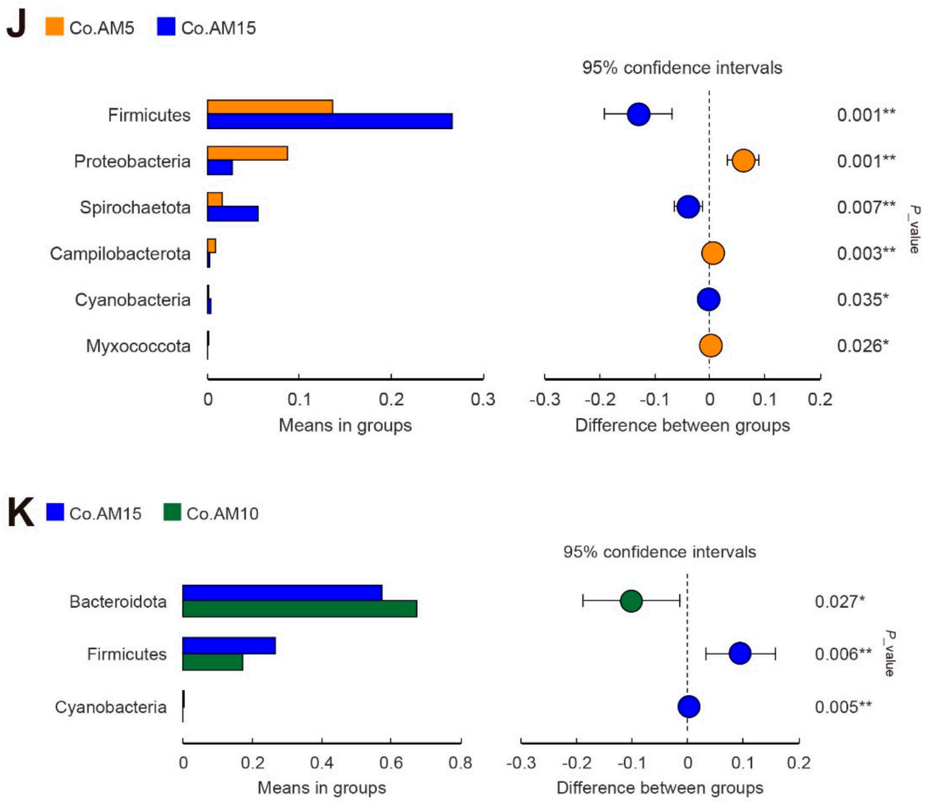

Figure S1. T-tests difference diagram at phylum level of (A-E) cecum and (F-K) colon microbiota under different alfalfa meal levels. Ce = cecum, Co = colon, AM = alfalfa meal.

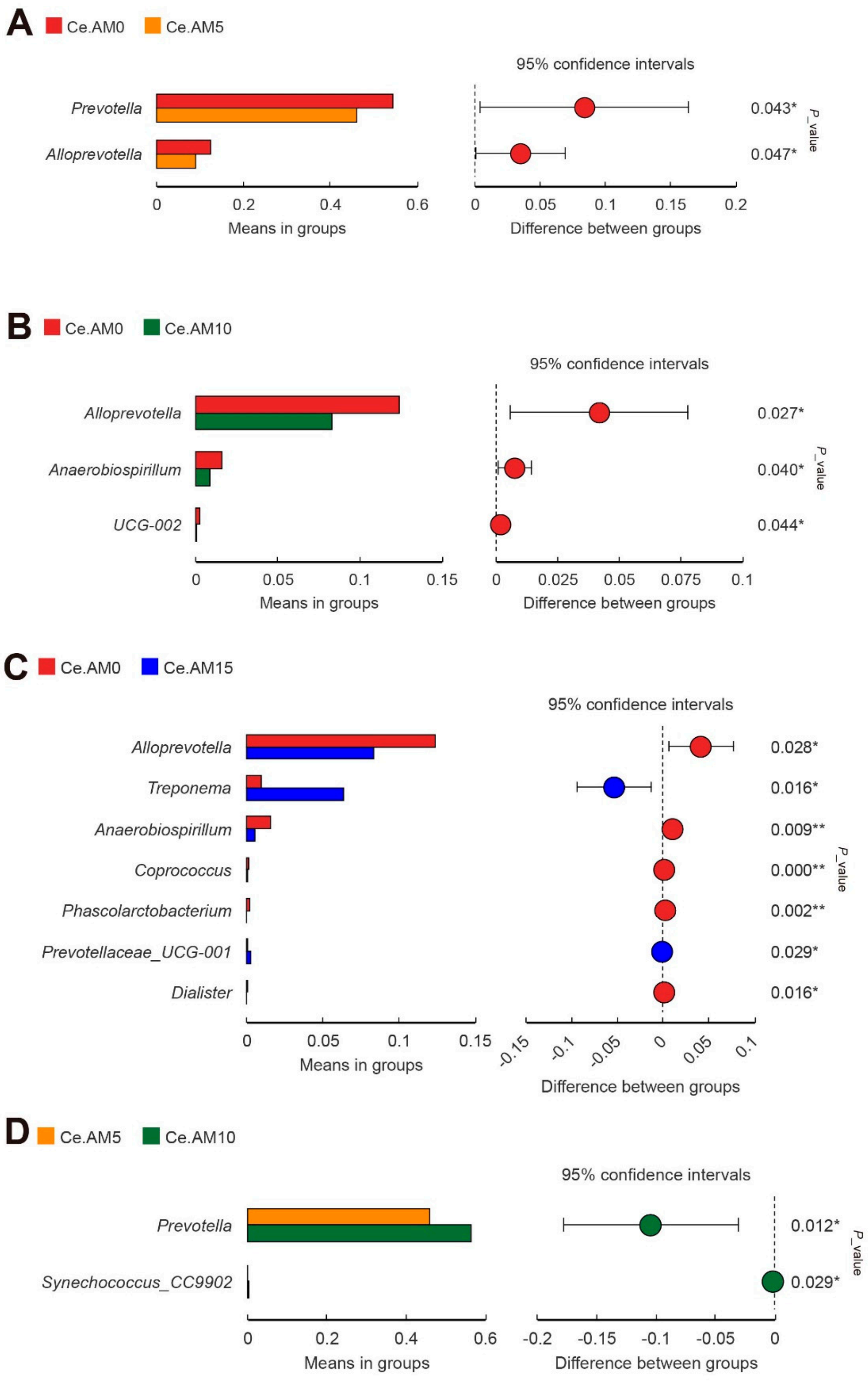
A
Ce.AM0
Ce.AM5
95% confidence intervals
Prevotella
Alloprevotella
0.043*
0.047*
P_value
0
0.2
0.4
0.6
Means in groups
0
0.05
0.1
0.15
0.2
Difference between groups
B
Ce.AM0
Ce.AM10
95% confidence intervals
Alloprevotella
Anaerobiospirillum
UCG-002
0.027*
0.040*
0.044*
P_value
0
0.05
0.1
0.15
Means in groups
0
0.025
0.05
0.075
0.1
Difference between groups
C
Ce.AM0
Ce.AM15
95% confidence intervals
Alloprevotella
Treponema
Anaerobiospirillum
Coprococcus
Phascolarctobacterium
Prevotellaceae_UCG-001
Dialister
0.028*
0.016*
0.009**
0.000**
0.002**
0.029*
0.016*
P_value
0
0.05
0.1
0.15
Means in groups
-0.15
-0.1
-0.05
0
0.05
0.1
Difference between groups
D
Ce.AM5
Ce.AM10
95% confidence intervals
Prevotella
Synechococcus_CC9902
0.012*
0.029*
P_value
0
0.2
0.4
0.6
Means in groups
-0.2
-0.15
-0.1
-0.05
0
Difference between groups

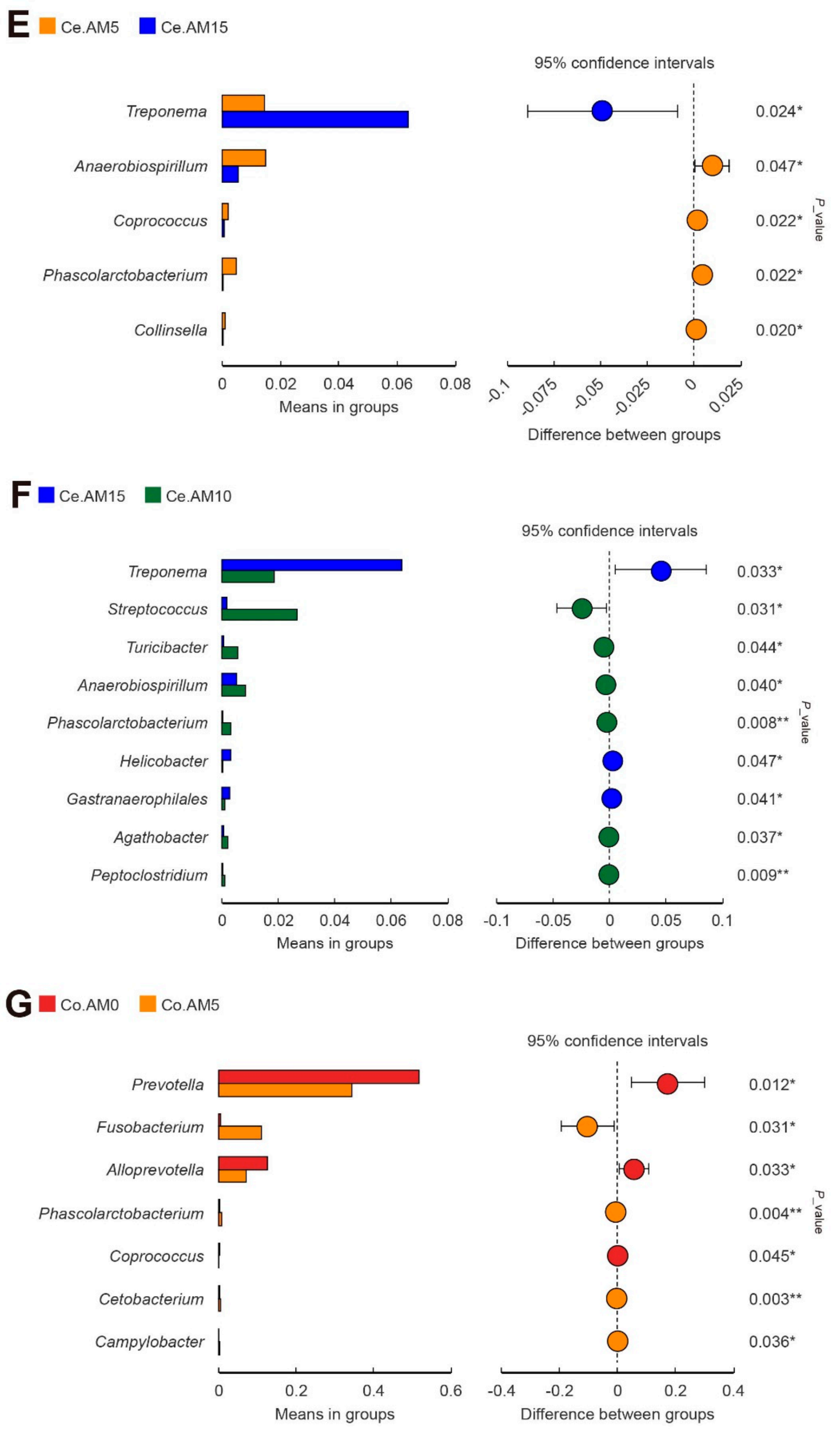
E
Ce.AM5
Ce.AM15
95% confidence intervals
Treponema
Anaerobiospirillum
Coprococcus
Phascolarctobacterium
Collinsella
0.024*
0.047*
0.022*
0.022*
0.020*
P_value
0 0.02 0.04 0.06 0.08
Means in groups
-0.1 -0.075 -0.05 -0.025 0 0.025
Difference between groups
F
Ce.AM15
Ce.AM10
95% confidence intervals
Treponema
Streptococcus
Turicibacter
Anaerobiospirillum
Phascolarctobacterium
Helicobacter
Gastranaerophilales
Agathobacter
Peptoclostridium
0.033*
0.031*
0.044*
0.040*
0.008**
0.047*
0.041*
0.037*
0.009**
P_value
0 0.02 0.04 0.06 0.08
Means in groups
-0.1 -0.05 0 0.05 0.1
Difference between groups
G
Co.AM0
Co.AM5
95% confidence intervals
Prevotella
Fusobacterium
Alloprevotella
Phascolarctobacterium
Coprococcus
Cetobacterium
Campylobacter
0.012*
0.031*
0.033*
0.004**
0.045*
0.003**
0.036*
P_value
0 0.2 0.4 0.6
Means in groups
-0.4 -0.2 0 0.2 0.4
Difference between groups

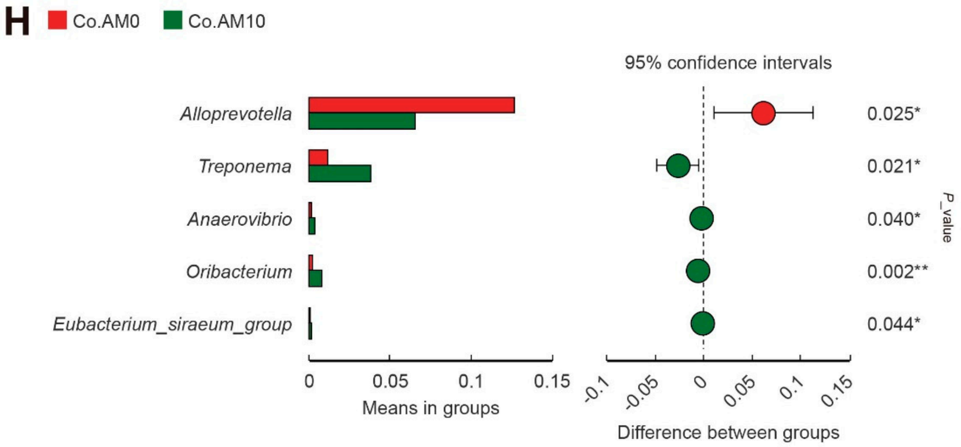
H
Co.AM0
Co.AM10
95% confidence intervals
Alloprevotella
Treponema
Anaerovibrio
Oribacterium
Eubacterium_siraeum_group
0.025*
0.021*
0.040*
0.002**
0.044*
P_value
0
0.05
0.1
0.15
Means in groups
-0.1
-0.05
0
0.05
0.1
0.15
Difference between groups

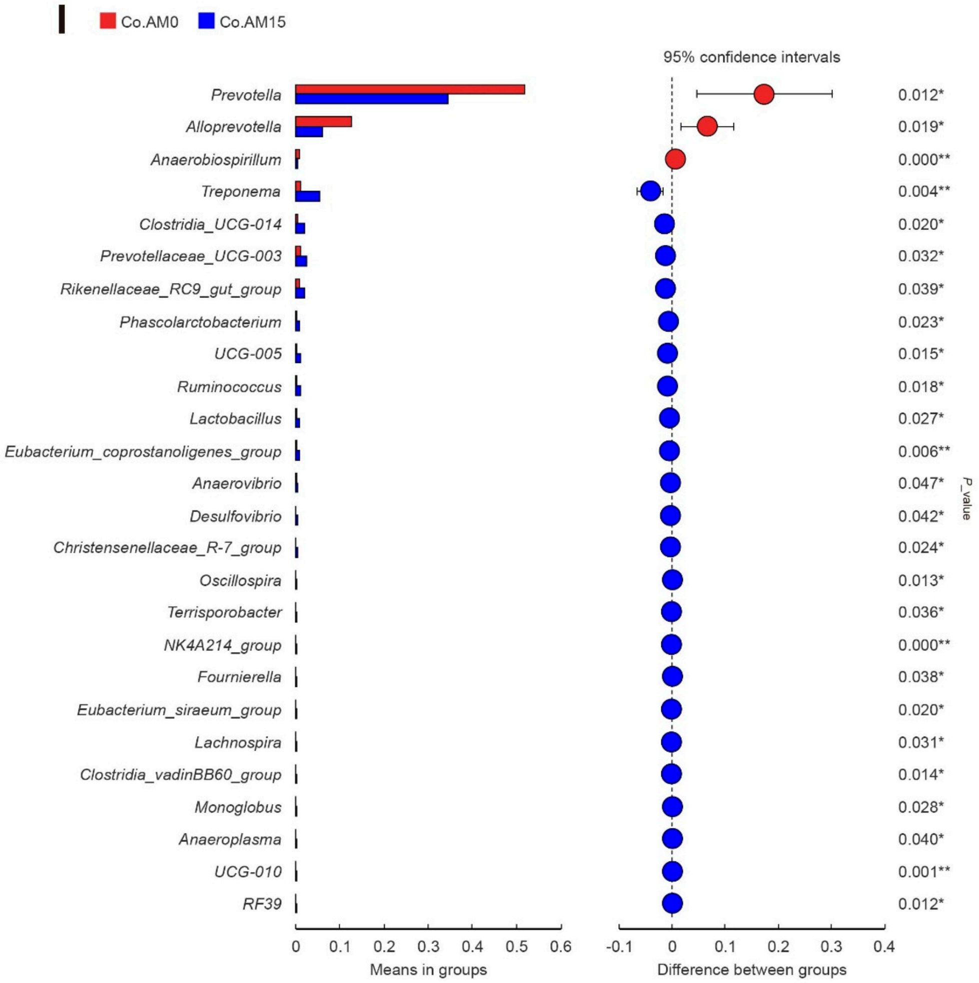
I
Co.AM0
Co.AM15
95% confidence intervals
Prevotella
Alloprevotella
Anaerobiospirillum
Treponema
Clostridia_UCG-014
Prevotellaceae_UCG-003
Rikenellaceae_RC9_gut_group
Phascolarctobacterium
UCG-005
Ruminococcus
Lactobacillus
Eubacterium_coprostanoligenes_group
Anaerovibrio
Desulfovibrio
Christensenellaceae_R-7_group
Oscillospira
Terrisporobacter
NK4A214_group
Fournierella
Eubacterium_siraeum_group
Lachnospira
Clostridia_vadinBB60_group
Monoglobus
Anaeroplasma
UCG-010
RF39
0.012*
0.019*
0.000**
0.004**
0.020*
0.032*
0.039*
0.023*
0.015*
0.018*
0.027*
0.006**
0.047*
0.042*
0.024*
0.013*
0.036*
0.000**
0.038*
0.020*
0.031*
0.014*
0.028*
0.040*
0.001**
0.012*
P_value
0
0.1
0.2
0.3
0.4
0.5
0.6
Means in groups
-0.1
0
0.1
0.2
0.3
0.4
Difference between groups

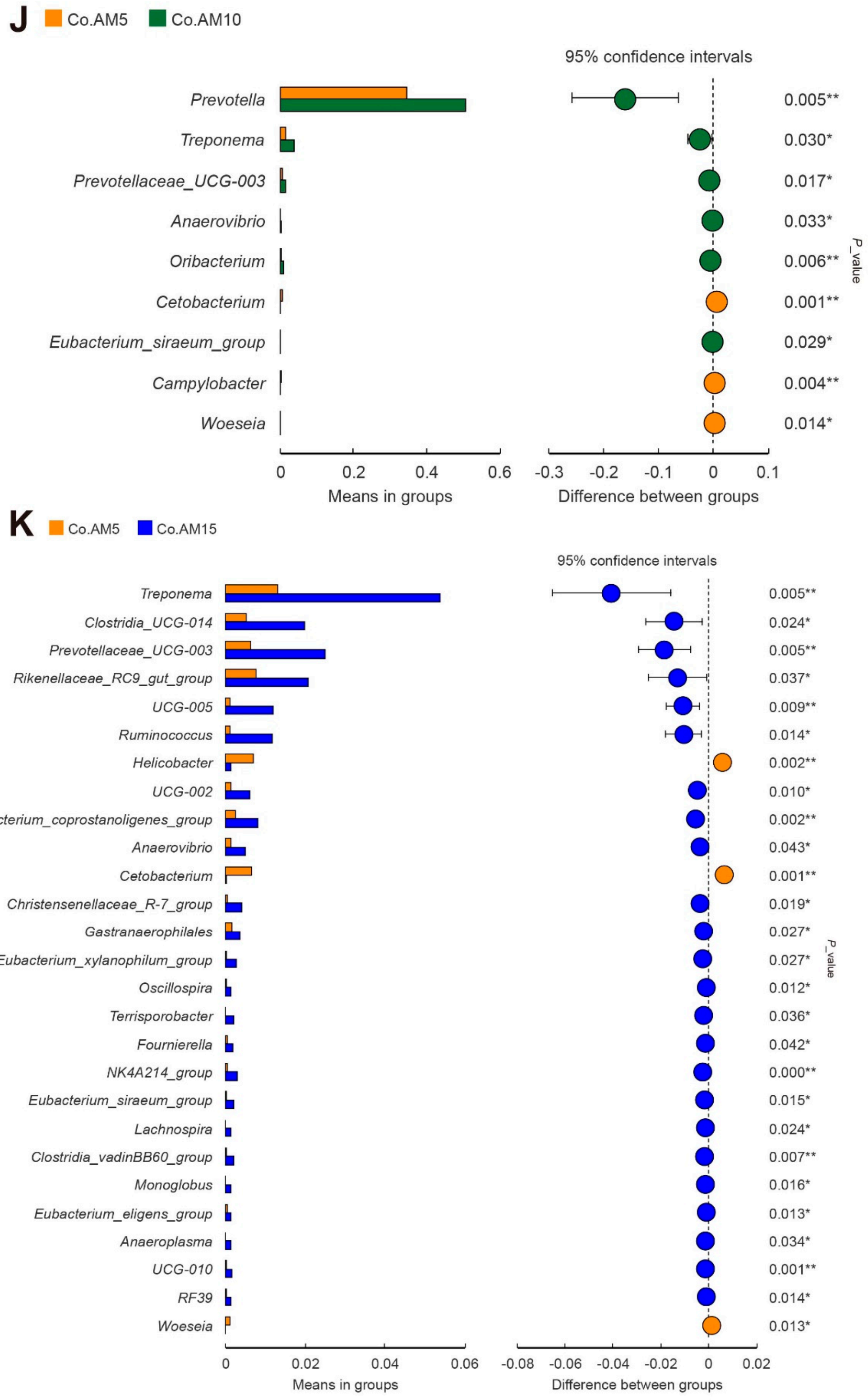
J
Co.AM5
Co.AM10
95% confidence intervals
Prevotella
Treponema
Prevotellaceae_UCG-003
Anaerovibrio
Oribacterium
Cetobacterium
Eubacterium_siraeum_group
Campylobacter
Woeseia
0.005**
0.030*
0.017*
0.033*
0.006**
0.001**
0.029*
0.004**
0.014*
P_value
0
0.2
0.4
0.6
Means in groups
-0.3
-0.2
-0.1
0
0.1
Difference between groups
K
Co.AM5
Co.AM15
95% confidence intervals
Treponema
Clostridia_UCG-014
Prevotellaceae_UCG-003
Rikenellaceae_RC9_gut_group
UCG-005
Ruminococcus
Helicobacter
UCG-002
Eubacterium_coprostanoligenes_group
Anaerovibrio
Cetobacterium
Christensenellaceae_R-7_group
Gastranaerophilales
Eubacterium_xylanophilum_group
Oscillospira
Terrisporobacter
Fournierella
NK4A214_group
Eubacterium_siraeum_group
Lachnospira
Clostridia_vadinBB60_group
Monoglobus
Eubacterium_eligens_group
Anaeroplasma
UCG-010
RF39
Woeseia
0.005**
0.024*
0.005**
0.037*
0.009**
0.014*
0.002**
0.010*
0.002**
0.043*
0.001**
0.019*
0.027*
0.027*
0.012*
0.036*
0.042*
0.000**
0.015*
0.024*
0.007**
0.016*
0.013*
0.034*
0.001**
0.014*
0.013*
P_value
0
0.02
0.04
0.06
Means in groups
-0.08
-0.06
-0.04
-0.02
0
0.02
Difference between groups

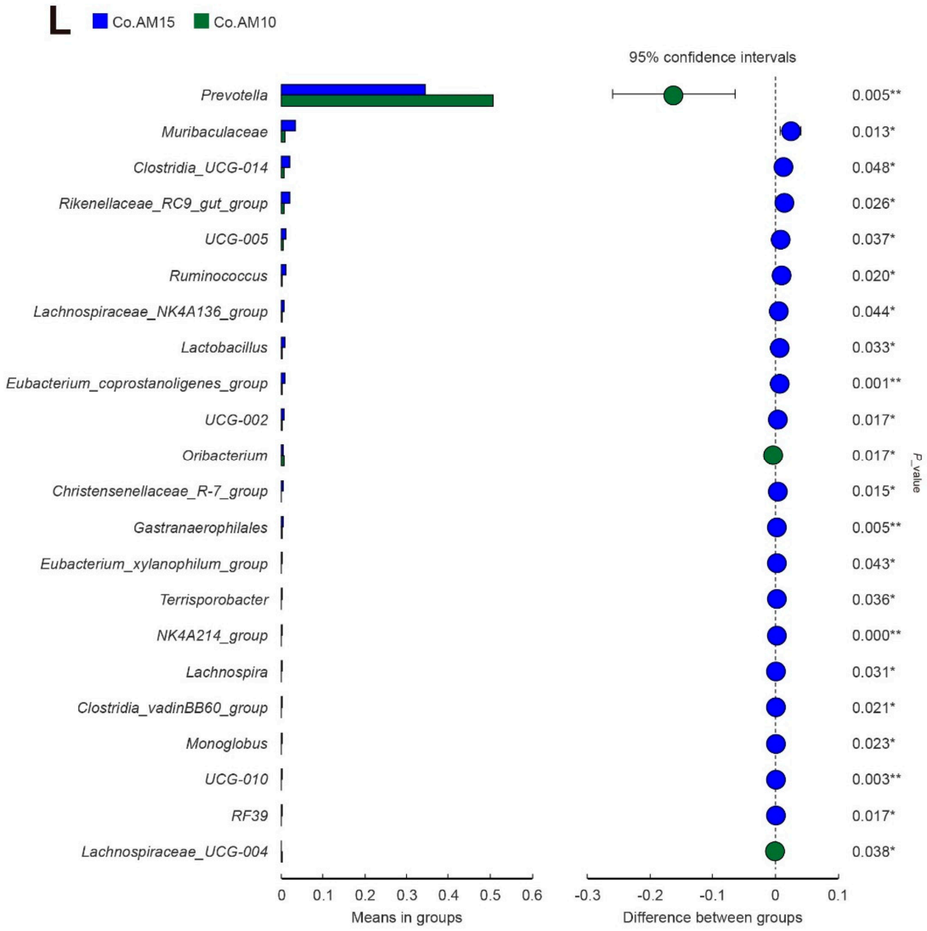

Figure S2. T-tests difference diagram at genus level of (A-F) cecum and (G-L) colon microbiota under different alfalfa meal levels. Ce = cecum, Co = colon, AM = alfalfa meal.
